# Kentucky Medical Society

**Published:** 1854-11

**Authors:** 


### KENTUCKY MEDICAL SOCIETY.

We had not the pleasure of being present at the last meeting of the Medical Society of Kentucky, which we understand was one of perfect harmony and of great interest. We copy from the *Frankfort Commonwealth* the following:—

*Official Report of the Proceedings of the Fourth Annual Meeting of the Kentucky State Medical Society, held in the City of Covington, on the 18th, 19th, and 20th of October*, 1854.

The Society met in the Presbyterian Church, and was opened with prayer by Rev. Mr. Morrell. The President, Dr. Gross, called the meeting to order at 3 o'clock, p. m. The roll was called and the minutes of last meeting read and adopted.

The following gentlemen were proposed for membership:—W. D. Holt, T. N. Wise, J. T. Wise, R. H. Hunt, C. Y. Lee, Covington; A. H. Johns, Independence; J. J. Dulaney, Covington; N. B. Shaler, Newport; F. Major, Covington; G. W. Thornton, J. Q. A. Foster, W. J. Sloan, Newport; J. T. Smith, S. S. Scott, Florence; W. H. Curran, Claysville; C. C. Bryson, Independence; E. Richardson, Monticello; S. R. Owen, Somerset.

On motion, Dr. Lawson, of Cincinnati, who was present, was invited to a seat with the Society. On motion of Dr. Chambers, the Society adjourned for a few moments. When called to order, it proceeded to the election of the above named gentlemen. They were unanimously elected.

### Special Reports.

The President called for reports from Special Committees, when Dr. Chipley handed in a report from Dr. Darby, of Lexington, Chairman of the Committee on the use of cold water as a Therapeutic agent in the treatment of disease. It was, on motion, laid on the table for the present.

Dr. Darby, at the same time, presented to the Society some pure vaccine virus, lately obtained by him from London, with printed directions for the proper method of using it; also, a request that it should be distributed in the cities of Louisville, Maysville, and Covington. The Society returned thanks for the present.

Dr. Reeves, of Cincinnati, being present was invited to a seat with, and to participate in the deliberations of the Society.

Dr. C. J. Blackburn, of Covington, by a dispensation of the rules of the Society, was unanimously elected a member.

The Society then adjourned to meet again at 7 o'clock, p. m., at the Methodist Episcopal Church to hear the Annual Address of the President.

---

7 o'clock, p. m.

The Society met according to adjournment, when the President proceeded to read the Annual Address; which on motion was received and referred to the Committee on Publication.

The Society then adjourned till to-morrow at 10 o'clock, a. m.

The address was listened to with marked attention by the audience, and the speaker succeeded in making a highly favorable impression in favor of the Society. The discourse commenced with a sketch of medical societies in Europe and in this country. Interesting statistics in relation to the importance of sanatory regulations, were introduced, and a recommendation that the Society should use its influence with the Legislature to procure a sanatory survey of the State. He also adverted to the importance of making some effort to obtain the appointment of medical men to assist in the examination of medical witnesses, in trials where skill in the profession would promote the ends of justice. In relation to the evils of quackery, which subsidized the press, the bar, and even the pulpit, to certify the wonders of its agency, it was neither to be put down by the medical press nor public discussion. The only remedy was to let it alone, and by the regular profession persevering in their devotion to the sick, and by the observance of their rights and privileges, and the purity of their objects. He also adverted to the rates of remuneration received by medical men, and inculcated attention to their pecuniary interests. Gratuitous services to the clergy he protested against as wrong in principle. If it originated in a love of charity, it might be tolerated, but with real charity and good feeling it had not the slightest sympathy. The importance of embodying the results of their experience in written productions which could be afterwards published for the benefit of the Society, the subject of epidemic disease, and a few appropriate remarks upon the death of one of the members of the Society,. Dr. E. C. DRANE, of Newcastle, closed the interesting address.

---

### SECOND DAY.

OCTOBER 19, 1854.

The Society met according to adjournment; the President in the chair. The minutes of the previous meeting read and adopted.

The Committee on Nominations reported the following as candidates for officers of the Society for the next year:

*President.*—Dr. C. H. SPILLMAN, Harrodsburg.

*Senior Vice President.*—Dr. A. EVANS, Covington.

*Junion Vice President.*—Dr. D. D. THOMSON, Louisville.

*Treasurer.*—Dr. B. M. WIBLE, Louisville.

*Recording Secretary.*—Dr. W. C. SNEED, Frankfort.

*Corresponding Secretary.*—R. J. BRECKINRIDGE, Louisville.

*Librarian.*—Dr. J. M. MILLS, Frankfort.

*Committee of Publication.*—T. S. BELL, T. G. RICHARDSON, JNO. BARTLETT, Louisville.

The report was received, and the committee discharged.

The following gentlemen were proposed for membership: Dr. P. L. REDDICK, Newport; Dr. DOWNARD, Independence.

The Society adjourned for a short time, and after being called to order proceeded to elect the above named candidates.

The President called for reports from Special Committees, whereupon one on Typhoid Fever, by Dr. SMITH, of Danville, was handed in, and on motion laid on the table for the present.

Dr. SPILLMAN, Chairman of the Committee on Mal-Practice, then read his report, which was received and referred to the Committee on Publication.

The following is a brief abstract of the report:

After some apologetic introductory remarks, it went on to say that "so unsettled and indeterminate are the premises, and so constant the alteration of lights and shadows in the field of medico-legal science, that the conclusions attainable in the present state of our knowledge are necessarily, to some extent, inconclusive and unsatisfactory."

Adverting to the antiquity of medical science, the report continued: "I would not disguise the fact that during the last half century, the relative position of our profession to the public has undergone a marked change. Implicit confidence, amounting in some instances to blind credulity, has given place to wide-spread skepticism as to the powers and capabilities of the healing art. For this, our profession is, in part, responsible, but not mainly so. The rage for speculation and theorising has had this tendency, when observation and experience have been neglected. Disparaging remarks about one member of the profession by another has the effect.

One of the principal causes of this skepticism, over which the profession can have no control, are want of information among the masses, relative to medical science, and a misconception of its powers leading to undue expectations. To remedy this want of information, medical statistics should be thrown broadcast over the land. Another reason for this skepticism is the apparent contrariety in the doctrines and practices of its different members. There may be apparent discrepancy, yet harmony in result; thus the lancet depletes, purgatives deplete, emetics deplete, and diaphoretics deplete."

Having thus reviewed the relations of the medical profession to the public, the report went on to show to what extent the former should be responsible to the civil tribunals.

In all cases a legal arbiter should be thoroughly acquainted with the subject matter of the offense. Generally, judge, jury and witnesses, in trials for mal-practice are profoundly ignorant of medical science. The physician should be amenable to law, but such judicial regulations should be adopted as will rescue the profession from the hazard of having its members subjected to citation and trial before a tribunal ignorant of the matter in controversy.

The remedy lies in a legal enactment that physicians charged with mal-practice, shall be tried by a jury of their peers, *i. e.* Doctors. A number of cases, showing in a strong light the necessity for this regulation, were stated in the report, and the danger of present modes driving good men from the profession, was urged. The report next discussed the character of those whose conduct should be regarded as a subject for adjudication. There should be an intent to do injury.

The want of skill goes to show such intent, or at least recklessness, and hence mal-practice may be, 1st. reckless, 2nd. willful, 3rd. negligent, 4th. ignorant. Each of these topics being minutely examined, and also under the last, mal-practice through intoxication, criticised, the report concluded with a brief and clear *resumé* of the train of argument.

ELECTION OF OFFICERS.

After the reading of Dr. SPILLMAN's report, the Society went into the election of officers. Dr. SPILLMAN having no opposition, was declared unanimously elected President for the ensuing year, and after being conducted to the chair returned thanks for the honor conferred in a few appropriate remarks.

On motion of Dr. HAWKINS, the rules were dispensed with and all the other candidates voted for at once. There being no opposition, the candidates nominated by the convention were all unanimously elected.

Dr. GROSS offered the following as an amendment to the constitution, which lies over for one day:

*Resolved*, That the Kentucky State Medical Society shall hereafter hold its annual meetings in the City of Frankfort on the first Wednesday in February of each year.

Dr BRADFORD, Chairman of the Committee on Ovariotomy, asked and obtained further time, until the next annual meeting, to make his report; when on motion, the Society adjourned until 2 o'clock, P. M.

---

2 O'CLOCK, P. M.

The Society was called to order by the President at the appointed hour. Dr. B. W. SOUTHGATE, of Boone county, and Dr. J. N. HUGHES, of Louisville, were proposed for membership; and after the usual course, were elected.

Further reports from Special Committees were called for, when Dr. Breckinridge, Chairman of the Committee on Medical Biography explained that he had made every effort to get his report ready for the present meeting, but owing to the difficulty of obtaining materials, he was compelled to ask further time, which was granted, with the assurance from Dr. B. that he would be ready at the next annual meeting.

Dr. Chambers obtained further time to make his report on substitutes for quinine.

Dr. Darby's report "on cold water as a therapeutic agent," was called for and read to the Society by Dr. Breckenridge. The report was received and referred to the Committee on Publication.

The following is a brief abstract of the report:

Therapeutic Agent.

Dr. B. commenced by stating the circumstances that first directed his attention thereto. He had read, on this subject, an essay by Dr. Cooke, and Currie's Medical Reports. The latter contains the testimony of Dr. Wright in favor of cold water in the treatment even of malignant fevers. In 1777 cold water was recommended by an English physician of high standing, and perhaps at a still earlier date. It is a powerful remedy, whose effects can be more readily determined than any other.

Dr. Cooks states that it was used both by Galen and Hippocrates. Dr. Forbes has written ably and lengthily in its favor. These facts are noticed to show that hydropathy did not originate with Presnitz, but was used by the regular faculty in conjunction with medicines.

The report stated in strong terms the efficacy of the cold dash, and the sufferings of patients burning up with fever, as described by M. Louis, and to whom water is refused.

Dr. Gregory tried the use of water in scarlet fever when his own children were attacked, and found it most effectual in conquering the disease. Why is not Dr. Gregory as good authority for the use of cold water, as on any other point? The object of this report is stated to be the re-introduction of the old practice of using water as a remedial agent.

The report concluded by describing a great number of cases in Dr. Darby's practice, where water had been used with the greatest advantage.

Dr. Smith's report on Typhoid Fever was then read by Dr. Breckinridge; received and referred to the Committee on Publication.

Dr. Smith commenced by remarking that the history, the diagnosis, and the other aspects of his subjects were ably and fully laid down in many medical works, and a repetition thereof would be a work of supererogation. His own observation of the disease had been limited, especially of typhus, and there was already an abundance of medical speculation upon other people's investigations, and lastly, it would be improper for him to discuss the epidemic character of typhoid fever, as this was specially the province of the Committee on Epidemics; he would therefore confine himself to making a few practical remarks and suggestions.

1st. I have found the first or onset symptoms, with but little variation, to be the same as those of all other eruptive affections, and often precisely the same as those of a bilious attack, and, therefore no one can certainly tell the nature of the disease simply from the symptoms at the onset. One may guess from the fact of the disease being in the neighborhood.

2d. I have always observed something like a regular remission, generally in the latter part of the night.

3d. I have for several years, invariably directed the use of stimulants during the remissions. Liking the effect of alcoholic stimulants better than quinine.

4th. The danger in the disease is generally proportioned to the frequency of the pulse.

5th. Patients generally, with a dry warm skin, were less dangerous than when there is a constitutional tendency to perspiration.

6th. Constant high fever is less dangerous than a congestive tendency.

I have generally followed the expectant plan, meeting the indications general or local, as they came up.

The report concluded by suggesting the importance of an accurate and thorough chemical analysis of the four great secretions and excretions thrown off by the kidneys, bowels, lungs, and skin, during the progress of fever, cholera, pneumonia, and other diseases, and expressing the belief that by this, physicians would be enabled to practice with more confidence.

There being no further reports from Special Committees, reports from Standing Committees were called for. There being no reports from these Committees, the Society proceeded to the transaction of miscellaneous business.

The first in order was the election of members to the American Medical Association.

The following gentlemen were put in nomination and elected: Dr. Bradford, Augusta; Dr. Chipley, Lexington; Dr. Shaler, Newport; Dr. Pretlow, Covington; Dr. Forsythe, Louisville; Dr. Darby, Lexington; Dr. Wible, Louisville; Dr. Southgate, Boone county; Dr. Thompson, Louisville; Dr. Jo. Smith, Danville; Dr. T. N. Wise, Covington.

The following gentlemen were elected honorary members of the Society: Dr. Cartwright, New Orleans; Dr. B. Welford, Virginia; Dr. L. C. Reeves, Cincinnati.

And then the Society adjourned until 10 o'clock to-morrow morning.

---

### THIRD DAY.

October 20, 1854.

The Society met pursuant to adjournment, the President in the chair.

The reading of the minutes of Thursday's proceedings were dispensed with.

Dr. Wible, Chairman of the Special Committee, appointed to memorialize the State Legislature, upon the subject of medical attendance at Coroner's inquests, and certain other grievances suffered by the profession, asked further time to present his memorial. This was granted.

Dr. Chambers asked further time to present a memorial upon the subject of patent medicines, and it was granted.

A letter from Dr. Sutton was received and read to the Society.

Dr. Chambers presented the following:

*Resolved*, That a Committee be appointed, whose duty it shall be to call the attention of the next Legislature, to a consideration of what is due to the State in a sanatory point of view, and they be instructed to propose a circular upon the subject, and transmit a copy of it to each Senator and Representative immediately after the next August election, and that said Committee be further instructed to press again upon the Legislature, the propriety of aiding in the publication of the transactions of this Society.

This resolution, after discussion, was adopted, and the President authorized to appoint the Committee with the other Special Committees.

Dr. Chambers offered a resolution that all Special Committees not reporting, or asking further time at the present session, be discontinued and the Chairman of Standing Committees be changed, as the President may see proper.

Dr. Wise objected that persons appointed on some Committees had made some preparations to report, but were prevented from being present at this time. He thought it would be more harmonious to let them remain. He stated that persons presenting excuses should be continued.

Dr. Wible amended the resolution to say that persons excusing themselves shall be continued.

Dr. Thompson explained the resolution as virtually including the amendment.

Several other gentlemen spoke, after which the resolutions were adopted.

Dr. Chambers moved the appointment of a Committee on Special Committees.

A resolution to that effect was passed, and Messrs. Chambers, Wible, and Pretlow, appointed on said Committee.

A letter was read from Dr. D. J. Ayres, regretting that a death in his family prevented his being present, and on motion, it was ordered on file.

The resolution amending the constitution so far as to determine on holding the sessions of the Society permanently in Frankfort, on the first Wednesday in February, of each year, was taken up, and after being discussed and amended so far as simply to declare that the next session of the Society should be held at that place on the first Wednesday of February, 1856, was passed.

On motion, Drs. Wm. Tibbatts and E. L. Grant were admitted to membership.

On motion of Dr. Thompson, it was ordered that the Secretary should send an abstract of the doings of this session to the Medical Journal, at Louisville, for publication.

The Committee on Special Committees, reported and were discharged.

The settlement of sundry claims for and against the Society, was discussed, and a Committee appointed to audit them.

The following Committees were appointed for the ensuing year:

STANDING COMMITTEES.

*Arrangements.*—Dr. Sneed.

*Public Hygiene.*—Dr. Thornton.

*Vital Statistics.*—Dr. John Swain.

*Epidemics.*—Dr. Chambers.

*Obstetrics.*—Dr. Letcher.

*Improvements in Practical Medicine.*—Dr. Moore.

*Improvements in Surgery.*—Dr. E. L. Dudley.

*Improvements in Pharmacy.*—Dr. Mills.

*Improvements in Botany.*—Dr. Emmert.

*Finance.*—Dr. Hewett.

*Publication.*—Drs. Bell, Bartlett, and T. G. Richardson.

SPECIAL COMMITTEES.

*Medical Biography.*—Dr. R. S. Breckinridge.

*Relations between Disease and Particular Geological Formations.*—Dr. Peter Lexington.

*Statistics of Lithotomy and Calculous Diseases.*—Dr. Gross.

*Results of Surgical Operations on Malignant Diseases.*—Dr. Colescott.

*Epidemic Dysentery.*—Dr. Patterson.

*Typhoid Fever.*—Dr. Southgate.

*Placenta Prævia.*—Dr. H. Miller.

*Scarlatina.*—Dr. Hawkins.

*Ovariotomy.*—Dr. Bradford.

*Inflammation and Ulceration of the Uterus.*—Dr. Foster.

*Physiological and Morbid Effects of Alcoholic drinks.*—Dr. Duke.

*Injuries of the Skull and Brain.*—Dr. Hardin.

*Anesthetics.*—Dr. B. Pretlow.

*Nervous Irritation.*—Dr. B. M. Wible.

*Plan to enable Physicians with more ease and certainty to collect their Accounts.*—Drs. J. N. Hughes, and R. J. Breckinridge.

*Intermittent Diseases.*—Dr. L. G. Ray.

*Remedies for Tubercular Diseases.*—Dr. T. N. Wise.

On motion, the Society adjourned temporarily, and after being called to orded, elected Drs. TIBBATTS and GRANT members, who had been previously nominated.

Dr. THOMPSON presented bills from Webb & Levering, of Louisville, for printing pıoceedings of second annual meeting; which bills, being a small balance, were referred to a Select Committee consisting of Drs. Thompson and Wible. A bill from the office of the *Louisville Democrat* for publishing notice of second meeting was referred to the same committee.

The Society then adjourned to meet at 3 o'clock, P. M.

---

3 O'CLOCK, P. M.

The Society met according to adjournment.

On motion, ordered that the committee to memorialize the Legislature on a sanitory survey of the State shall consist of seven members, as follows: Dr. Sutton, Georgetown; Dr. Chipley, Lexington; Dr. Sneed, Frankfort; Dr. Bell, Louisville; Dr. Chambers, Covington; Dr. Duke, Maysville; Dr. Smith, Danville.

On motion, the Secretary was directed to have the proceedings of this meeting published in the *Frankfort Commonwealth*, at the expense of the Society, and to send a copy to each member.

A resolution of thanks was passed to the members of the Fourth Presbyterian church, for the use of the church during the session of the Society.

A resolution of thanks was also tendered to the Trustees of the Methodist Episcopal church for the use thereof, for the delivery of the annual address.

Thanks were also tendered to the citizens and physicians of Covington, for kind entertainment, and to Dr. PRETLOW, of the Committee of Arrangements, for his services.

The Society then adjourned to meet at the banquet in the evening, and in the city of Frankfort on the first Wednesday in February, 1856.

C. H. SPILLMAN, *President.*

W. C. SNEED, *Secretary.*

Note.—If the members of the Society will forward the amount of their respective dues to the Secretary, at Frankfort, or the Treasurer, Dr. Wible, at Louisville, the Committee of Publication will be able, with the aid expected from individual members of the Society, to publish the proceedings of the two last meetings in the course of a very short time.

W. C. SNEED, *Secretary.*

